# Bilateral molar extraction during growth is associated with impaired masticatory performance and reduced hippocampal BDNF and TrkB immunoreactivity in juvenile rats

**DOI:** 10.1016/j.jobcr.2026.101553

**Published:** 2026-10-08

**Authors:** Harun Achmad, Dharli Syafriza, Sri Ratna Rahayu, Harlina, Andi Annisa Eka Aprilda, Andi Aliyah Nurul Syaikah, Nurul Mutmainnah, Fitri Apriliani, Yunita Dyah Puspita Santik, Harlina, Hans Lesmana, Rini Sitanaya

**Affiliations:** a Department of Pediatric Dentistry, Faculty of Dentistry, Hasanuddin University, Makassar, Indonesia; b Syiah Kuala University, Banda Aceh, Aceh, Indonesia; c Semarang State University, Semarang, Central Java, Indonesia; d Department of Oral Medicine, Faculty of Dentistry, Hasanuddin University, Makassar, Indonesia; e Department of Dental Health Polytechnic of Health Ministry Makassar, Indonesia

**Keywords:** Brain-derived neurotrophic factor, Hippocampus, Masticatory performance, Posterior tooth loss, Tropomyosin receptor kinase B, Wistar rat

## Abstract

**Background:**

Bilateral molar extraction during growth produces severe posterior occlusal deprivation and may impair mastication while altering oral sensorimotor input relevant to hippocampal function. Brain-derived neurotrophic factor (BDNF) and tropomyosin receptor kinase B (TrkB) contribute to hippocampal plasticity.

**Objective:**

To compare masticatory performance and hippocampal BDNF and TrkB immunoreactivity between juvenile rats with intact dentition and rats undergoing bilateral extraction of all maxillary and mandibular molars, and to explore pooled associations among these outcomes.

**Methods:**

Twenty-four juvenile male Wistar rats were enrolled; 17 reached the endpoint (control, n = 9; extraction, n = 8). Masticatory performance was measured using a granulometric masticatory performance index (MPI). BDNF and TrkB immunoreactivity in CA1, CA3, and dentate gyrus were assessed immunohistochemically. Between-group differences were analyzed using Welch t tests, 95% confidence intervals, and Hedges’ g. Pooled associations were examined using Pearson correlations.

**Results:**

The extraction group showed lower MPI (0.241 ± 0.043 vs 0.566 ± 0.044), BDNF scores (3.42 ± 0.64 vs 6.74 ± 1.52), and TrkB H-scores (86.86 ± 12.76 vs 180.73 ± 22.55); all p < 0.001. Pooled MPI correlated positively with BDNF (r = 0.783) and TrkB (r = 0.899), both p < 0.001, although these associations may largely reflect between-group separation.

**Conclusion:**

Bilateral molar extraction during growth was associated with impaired food comminution and lower hippocampal BDNF and TrkB immunoreactivity. These exploratory correlations do not establish mediation, within-group biological relationships, or causality. Attrition, absence of sham controls, and unmeasured postoperative confounding limit mechanistic interpretation in this model.

## Graphical abstract

**Figure:**
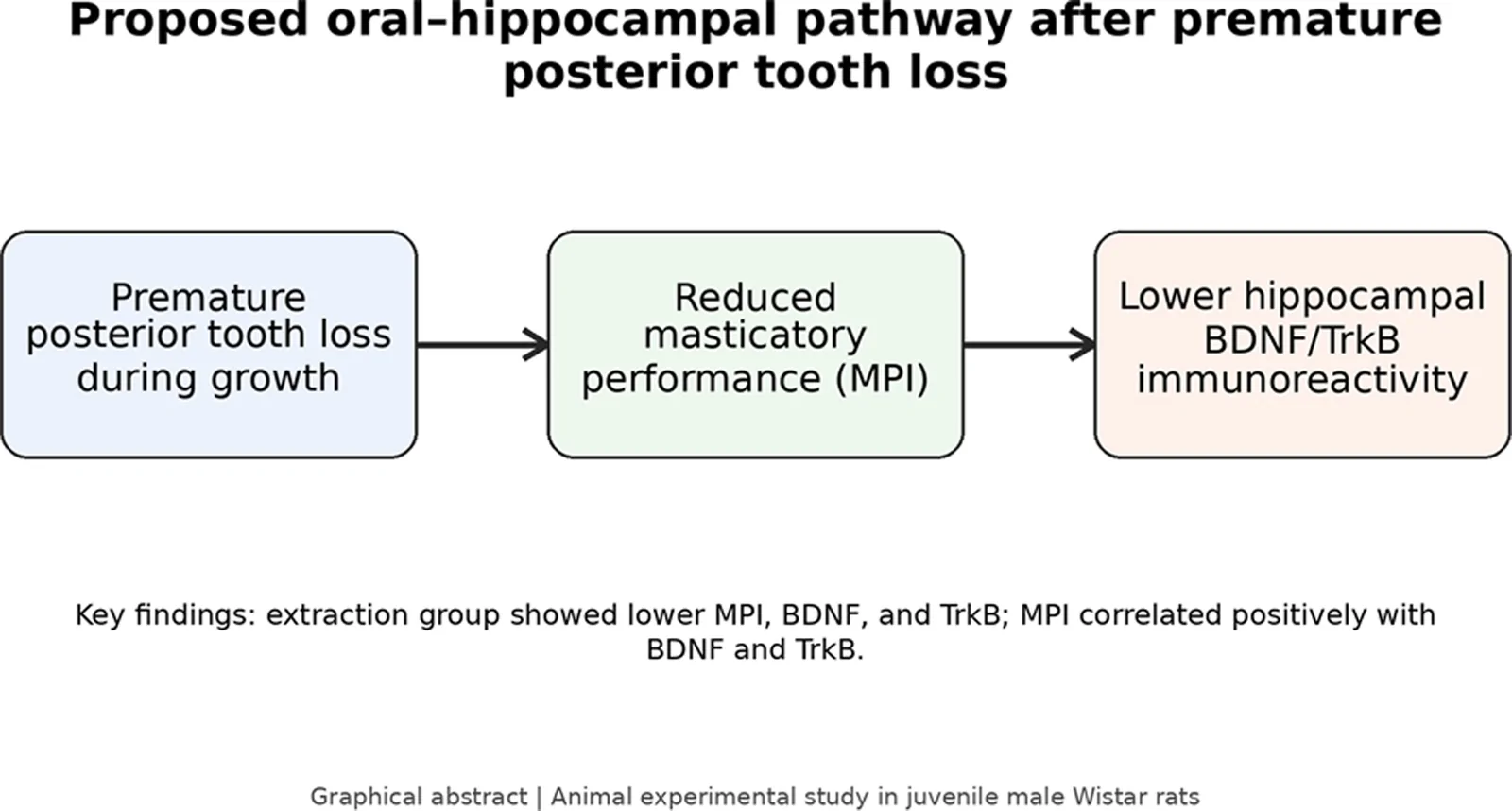

## 1 Introduction

Premature loss of posterior teeth during growth is clinically important because posterior teeth provide occlusal support, maintain arch integrity, and enable efficient food comminution. In pediatric dentistry, early loss of primary molars is most commonly associated with untreated caries, although trauma, periodontal disease, developmental abnormalities, and iatrogenic factors may also contribute.^1^, ^2^, ^3^, ^4^, ^5^ The local consequences include arch-length reduction, mesial migration, crowding, altered eruption, malocclusion, and impaired oral function.^2^^,^^5^

Mastication is a rhythmic sensorimotor behavior involving teeth, periodontal mechanoreceptors, oral mucosa, masticatory muscles, temporomandibular proprioception, salivary function, and brainstem motor pattern generation.^6^^,^^7^ Masticatory performance can be quantified objectively using granulometric methods based on the particle-size distribution of comminuted food.^7^

Human and animal evidence suggests that altered mastication may influence hippocampal circuits involved in learning and memory.^8^, ^9^, ^10^, ^11^, ^12^, ^13^ Experimental soft-diet feeding or molar extraction has been associated with changes in hippocampal morphology, neurogenesis, synaptic markers, neurotrophic signaling, and memory-related behavior.^8^, ^9^, ^10^, ^11^^,^^13^ However, many studies infer masticatory dysfunction from tooth loss or diet texture without measuring masticatory performance in the same animals.

BDNF and TrkB provide a biologically relevant activity-dependent signaling pathway. BDNF contributes to neuronal survival, differentiation, synaptogenesis, long-term potentiation, and memory-related synaptic consolidation.^14^, ^15^, ^16^, ^17^ Binding of mature BDNF to TrkB activates MAPK/ERK, PI3K/Akt, and PLC-gamma signaling pathways that support neuronal survival and CREB-dependent transcription.^16^, ^17^, ^18^ Nevertheless, reduced immunoreactivity does not by itself establish reduced receptor activation or downstream signaling.

The present study specifically evaluated the neurotrophic domain of the hippocampal response to severe posterior occlusal deprivation by comparing objectively measured masticatory performance and hippocampal BDNF and TrkB immunoreactivity between growing rats with intact dentition and rats undergoing bilateral molar extraction. The primary objective was to compare masticatory performance and BDNF/TrkB immunoreactivity between groups. A secondary, exploratory objective was to examine pooled associations between masticatory performance and the two neurotrophic outcomes. These analyses are exploratory and hypothesis-generating and are not designed to establish a mechanistic oral–brain pathway. For transparency, this manuscript represents the BDNF/TrkB neurotrophic analysis of a broader parent animal experiment; a companion report from the same experimental cohort (JOBCR-D-26-00607R1) evaluates distinct GFAP and synaptophysin outcomes, with the shared cohort and animal-level MPI dataset disclosed in Methods Section 2.1.

## 2 Materials and methods

### 2.1 Study design, reporting, and ethical approval

This laboratory animal experiment used a post-test-only control-group design and was prepared with reference to the ARRIVE 2.0 reporting recommendations.^19^ The protocol was approved by the Ethics Committee of the Faculty of Dentistry, Hasanuddin University Dental Hospital, Hasanuddin University (No. 298/KEPK-FKG-RSGMP-UH/EA/X/2025). The individual animal was the experimental unit. All procedures followed the approved institutional protocol and the principles of replacement, reduction, and refinement.

For transparency, this report represents the BDNF/TrkB neurotrophic analysis of a broader parent animal experiment. A companion report from the same experimental cohort (JOBCR-D-26-00607R1) evaluates distinct astroglial and presynaptic biomarker outcomes (GFAP and synaptophysin). The animal cohort, bilateral molar-extraction intervention, the same animal-level masticatory performance index (MPI) dataset, and hippocampal tissue collection are shared across the reports. MPI is retained in the present manuscript because it provides the functional phenotype against which the BDNF/TrkB outcomes are interpreted. GFAP and synaptophysin outcomes are not analyzed or reported in the present manuscript.

### 2.2 Animals and housing

Twenty-four male Wistar rats (*Rattus norvegicus*) were obtained at 3 weeks of age (approximately 90-100 g) from an animal breeding facility in Takalar, South Sulawesi, Indonesia, with accompanying health certification. Animals were acclimatized for 7 days; therefore, extraction was performed at approximately 4 weeks of age. Animals were housed under a 12-h light/dark cycle at 22 ± 2 °C and 50 ± 10% relative humidity, with adequate ventilation and standard cage density. Standard pellet chow (AD II Comfeed, PT Japfa Comfeed Indonesia Tbk, Indonesia) and water were provided ad libitum during acclimatization. Animals were observed daily for illness, distress, reduced activity, abnormal feeding, and weight loss.

### 2.3 Sample-size rationale

A conventional a priori power calculation was not performed because no directly comparable estimate was available for the prespecified granulometric primary outcome. The planned sample size was instead based on the resource-equation approach for exploratory animal experiments.^20^, ^21^, ^22^, ^23^ Using E = N - k, where E is the error degrees of freedom, N is the total number of animals, and k is the number of groups, a planned analytical total of 20 animals across two groups produced E = 18, within the recommended range of 10-20. The enrollment target was increased to 12 animals per group (24 total) to allow for anticipated attrition. The resource equation is not a substitute for a power calculation and does not guarantee adequate precision; this is acknowledged as a limitation.

### 2.4 Group allocation, concealment, and blinding

Animals were assigned at the animal level to the intact-dentition control group or the posterior-tooth-extraction group. Samples collected for MPI assessment and histological slides were labeled with coded identifiers before outcome assessment. Blinding of the operator performing extraction and personnel providing postoperative care was not feasible because the intervention was evident.

### 2.5 Posterior tooth extraction and postoperative care

On study day 8, animals assigned to extraction received ketamine 80 mg/kg and xylazine 8 mg/kg intraperitoneally. After loss of response to a noxious stimulus, all maxillary and mandibular molars were extracted bilaterally using standardized small-animal dental instruments and gentle luxation under aseptic conditions. Hemostasis was achieved with sterile gauze compression. Meloxicam was administered subcutaneously at 2 mg/kg immediately after extraction, followed by 1 mg/kg every 24 h for 2 additional days. Animals were monitored until full anesthetic recovery and then observed for bleeding, infection, reduced feeding, weight loss, and distress. A soft diet was provided to extraction animals for 3 postoperative days, after which standard pellet chow was resumed. Control animals did not undergo anesthesia, sham surgery, or extraction and continued to receive standard pellet chow and water ad libitum.

Because the control group did not undergo anesthesia or sham extraction and did not receive the temporary postoperative soft diet, effects specifically attributable to tooth loss cannot be fully separated from anesthesia, surgery, pain, tissue injury, and early dietary adaptation.

### 2.6 Masticatory performance assessment

MPI was assessed on study days 11, 12, and 13. Animals were fasted for 3 h before each test while retaining free access to water. Each animal was placed in a test cage fitted with a grid to prevent food particles from mixing with bedding and received a standardized 5-g sample of dry homogeneous pellet chow for 10 min. Food residue that was not swallowed, was expelled, or fell through the grid was collected using a fine brush and plastic forceps into coded Petri dishes. Residues were dried at 60 °C for 1 h or until constant weight, sieved through a 1-mm mesh using a sieve shaker for 1 min, and weighed on an analytical balance accurate to 0.001 g. MPI was calculated as the dry weight of particles ≤1 mm divided by the total dry residue weight. Values were expressed as proportions from 0 to 1, with higher values indicating better comminution. The mean of the three consecutive assessments was used for each animal.

### 2.7 Tissue collection and immunohistochemistry

On study day 22, corresponding to 14 days after extraction, animals were deeply anesthetized with a terminal ketamine-xylazine regimen and euthanized by cardiac puncture and exsanguination according to the approved protocol. Brains were removed, rinsed, and fixed in 10% neutral buffered formalin for 24-48 h. Tissue was dehydrated through graded ethanol, cleared in xylene, embedded in paraffin, and sectioned coronally at 4-6 μm to include the hippocampus.

Sections were deparaffinized and rehydrated. Heat-mediated antigen retrieval was performed in citrate buffer (pH 6.0). Endogenous peroxidase was blocked using 3% hydrogen peroxide for 10 min, and nonspecific binding was blocked using 5% normal goat serum for 30 min at room temperature. BDNF staining used a rabbit polyclonal anti-BDNF antibody (Affinity Biosciences, DF6387; 1:100) for 1.5 h at 22 °C. TrkB staining used an anti-TrkB antibody (Affinity Biosciences, AF6461). HRP-conjugated goat anti-rabbit secondary antibody, 3,3′-diaminobenzidine visualization, and Mayer hematoxylin counterstaining were used. Negative controls were processed without primary antibody.

### 2.8 Image acquisition and immunohistochemical scoring

Images were acquired using an Olympus CX23 light microscope equipped with a digital camera at 400× magnification under standardized illumination. At least three nonoverlapping fields were selected from each hippocampal subregion—CA1, CA3, and the dentate gyrus (DG)—using a systematic random-sampling approach. Histological slides were assigned coded identifiers before assessment, and two independent observers evaluated the selected fields while blinded to group allocation.

BDNF immunoreactivity was evaluated using a study-specific modified semi-quantitative scoring method. Field-level observations were categorized binarily as absent or negative (score 0) and present or positive (score 1). The original analytical dataset contained subregional BDNF scores on an observed 0-10 scale. For each hippocampal subregion, scores from the predefined fields and the two blinded observers were averaged, and the final hippocampal BDNF composite score for each animal was calculated as the mean of the CA1, CA3, and dentate gyrus scores.

BDNF composite score = (CA1 score + CA3 score + DG score)/3.

The available study documentation did not preserve the exact algorithm used to convert the field-level binary observations into the reported 0-10 subregional scores. Accordingly, the exact transformation cannot be independently reconstructed or reproduced from the retained records, and no unverified scoring formula has been introduced. The BDNF results are therefore treated as exploratory semi-quantitative observations.

TrkB immunoreactivity was quantified using an H-score that incorporated staining intensity and the proportion of immunoreactive cells. Staining intensity was categorized as absent (0), weak (1), moderate (2), or strong (3), and the H-score was calculated as:

H-score = Σ(Pi × i),

where *Pi* represents the percentage of cells within each staining-intensity category and *i* represents the corresponding intensity score. The theoretical H-score ranged from 0 to 300. Scores from the predefined fields, hippocampal subregions, and two observers were averaged to obtain the final TrkB score for each animal.

Interobserver reliability was not formally quantified using an intraclass correlation coefficient. The scores assigned by the two blinded observers were averaged for the final analysis.

### 2.9 Statistical analysis

Analyses were performed using IBM SPSS Statistics for Windows, version 26.0 (IBM Corp., Armonk, NY, USA). Continuous variables are reported as mean ± standard deviation. Distributional assumptions were evaluated using Q-Q plots and the Shapiro-Wilk test. Between-group differences were analyzed using Welch independent-samples t tests to provide robustness to unequal variances and unequal final group sizes. Results are reported as absolute mean differences with 95% CIs. Standardized effects are reported as Hedges' g with approximate 95% CIs because Cohen's d is upwardly biased in small samples. Pearson coefficients with 95% CIs were used for exploratory pooled correlations between MPI and BDNF or TrkB. Scatterplots were inspected for linearity and influential observations. All tests were two-sided, with p < 0.05 considered statistically significant. Analyses used complete cases; no missing values were imputed and animals that did not reach the endpoint were not replaced.

## 3 Results

### 3.1 Animal flow and attrition

Twenty-four animals were initially allocated to the control and extraction groups, with 12 animals in each group. During the 7-day acclimatization period, three animals were lost: one from the control group and two from the extraction group. Four additional animals did not reach the experimental endpoint, comprising two animals from the control group and two from the extraction group. Consequently, the final analysis included 17 animals: nine controls and eight extraction animals. No replacement animals were introduced. Detailed contemporaneous records regarding the clinical signs, exact causes of death, supportive treatment, necropsy findings, and attainment of predefined humane endpoints were not available for all animals. Therefore, the causes of the losses could not be definitively established.

### 3.2 Between-group comparisons

The extraction group had markedly lower masticatory performance and lower hippocampal BDNF and TrkB immunoreactivity than the control group (Table 1; Fig. 1, Fig. 2).

**Table 1:** Between-group comparisons of masticatory performance and hippocampal immunoreactivity. Mean differences are calculated as control minus extraction. CI, confidence interval; MPI, masticatory performance index; SD, standard deviation. Welch independent-samples t tests were used.

Outcome	Control, n = 9 mean ± SD	Extraction, n = 8 mean ± SD	Mean difference (95% CI)	p value	Hedges' g (95% CI)
MPI	0.566 ± 0.044	0.241 ± 0.043	0.325 (0.280-0.370)	<0.001	7.09 (4.39-9.78)
BDNF composite score	6.74 ± 1.52	3.42 ± 0.64	3.32 (2.10-4.54)	<0.001	2.64 (1.33-3.95)
TrkB H-score	180.73 ± 22.55	86.86 ± 12.76	93.87 (74.91-112.83)	<0.001	4.78 (2.85-6.72)

**Fig. 1:**
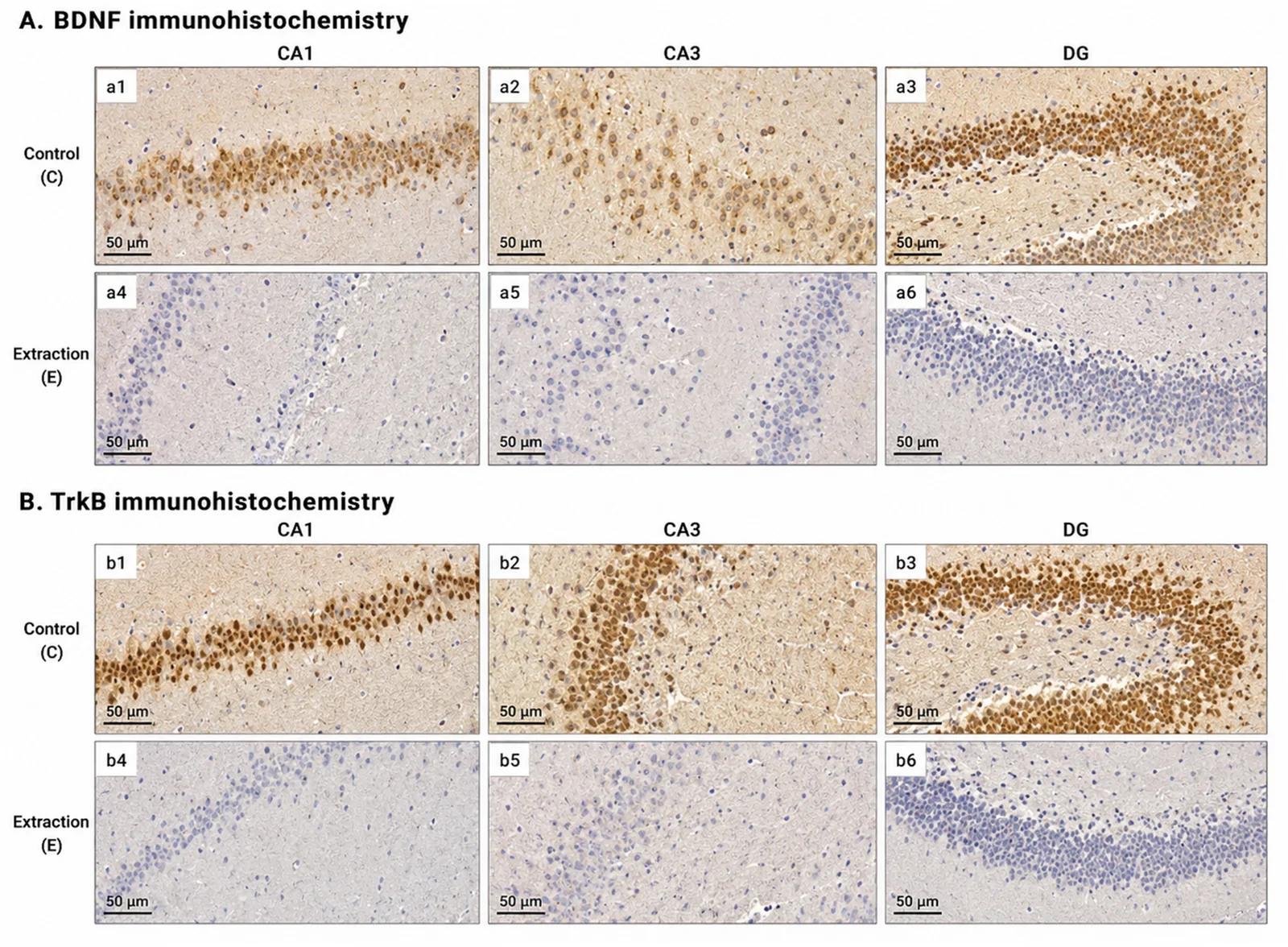
Representative immunohistochemical staining of brain-derived neurotrophic factor (BDNF) (A) and tropomyosin receptor kinase B (TrkB) (B) in the hippocampal CA1, CA3, and dentate gyrus (DG) regions. C, control group; E, extraction group. Brown 3,3′-diaminobenzidine (DAB) staining indicates positive immunoreactivity. All photomicrographs were acquired at 400× magnification under standardized illumination. Any brightness and contrast adjustments were applied uniformly across all panels. Scale bar = 50 μm. (For interpretation of the references to colour in this figure legend, the reader is referred to the Web version of this article.)

**Fig. 2:**
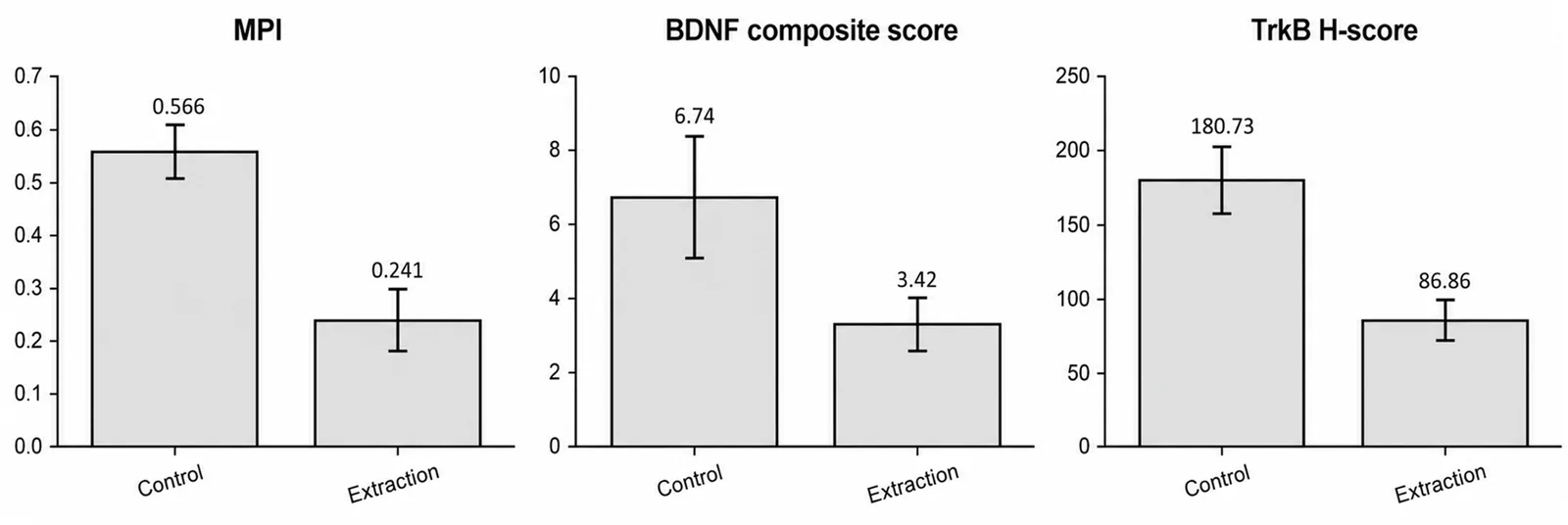
Group means and standard deviations for MPI, BDNF composite score, and TrkB H-score. Outcomes are displayed on separate axes because the BDNF and TrkB scores have different scales and should not be directly compared numerically.

The standardized effects were exceptionally large. These estimates arose from substantial absolute between-group separation combined with limited within-group dispersion. Their magnitude should not be interpreted as a precise population estimate because the final groups were small, immunohistochemical outcomes were semi-quantitative, and differential attrition may have selected nonrepresentative survivors.

### 3.3 Exploratory pooled correlations

In the pooled sample, MPI correlated positively with BDNF and TrkB immunoreactivity (Table 2; Fig. 3). However, visual inspection showed clear separation between the control and extraction groups. The pooled correlations may therefore predominantly reflect between-group separation rather than within-group covariation. Within-group correlations were not examined because the small final group sizes (control, n = 9; extraction, n = 8) provided insufficient statistical power for reliable within-group correlation estimates.

**Table 2:** Exploratory pooled correlations between masticatory performance and hippocampal immunoreactivity. CI, confidence interval; MPI, masticatory performance index.

Variables	N	Pearson r	95% CI	p value
MPI and hippocampal BDNF composite score	17	0.783	0.485-0.918	<0.001
MPI and hippocampal TrkB H-score	17	0.899	0.737-0.963	<0.001

**Fig. 3:**
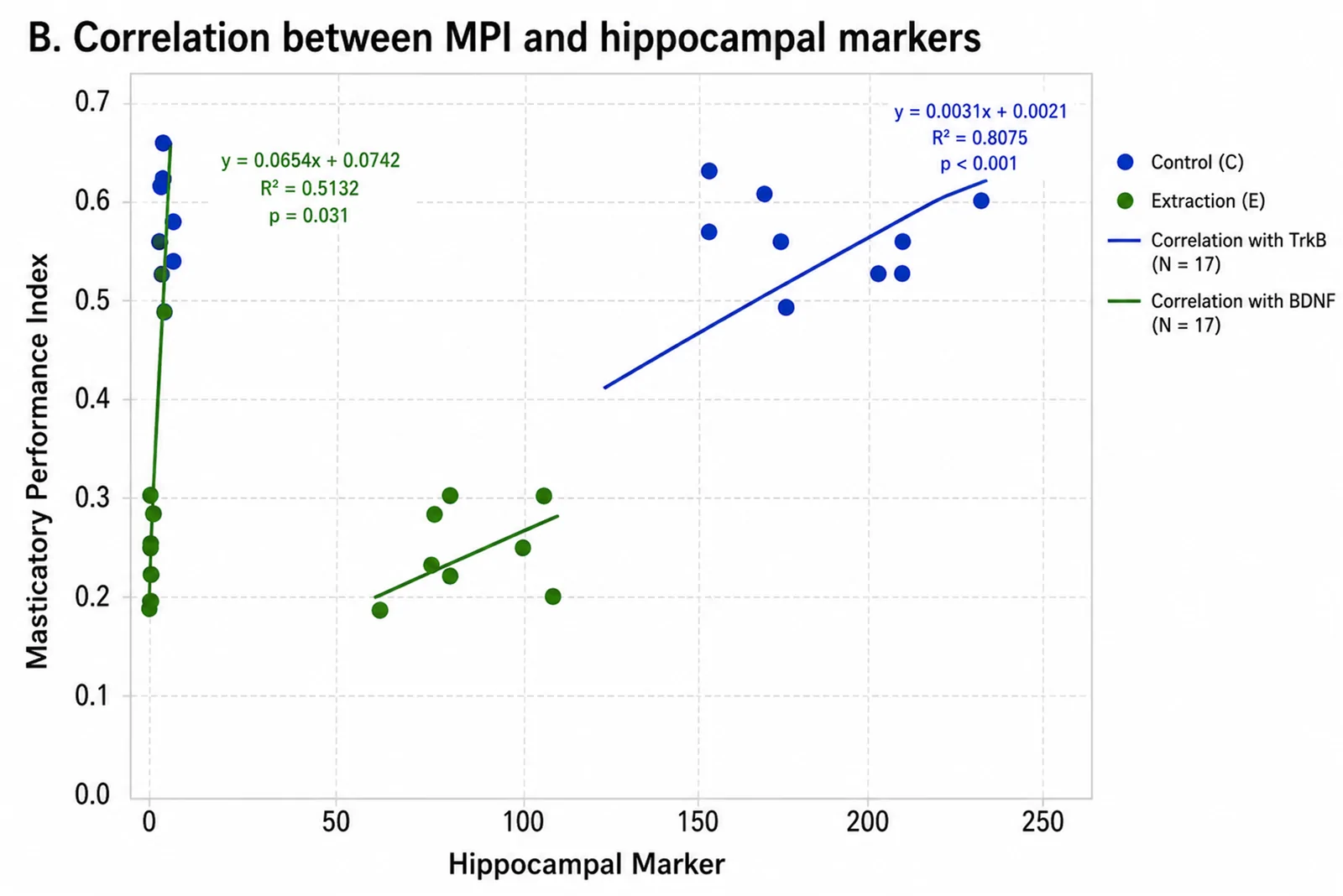
Scatterplots of the pooled associations between MPI and hippocampal BDNF or TrkB immunoreactivity. Points are distinguished by experimental group. Pooled regression lines are shown for visualization only; inferential statistics are reported in Table 2. The pooled associations may largely reflect between-group separation and should not be interpreted as evidence of mediation or within-group causality.

## 4 Discussion

This exploratory, hypothesis-generating preclinical study found that bilateral molar extraction during growth was associated with markedly impaired food comminution and lower hippocampal BDNF and TrkB immunoreactivity. A principal strength of the study was the simultaneous assessment of an objective oral functional outcome and hippocampal molecular markers in the same animals. Previous animal studies commonly used molar extraction, molarless conditions, or soft-diet feeding as proxies for reduced mastication.9–13,24–26 Direct measurement of the Masticatory Performance Index (MPI) provided functional confirmation that the extraction intervention substantially impaired food comminution. The present report is intentionally restricted to the BDNF/TrkB neurotrophic domain of the parent experiment.

The profound reduction in MPI is consistent with the mechanical role of posterior teeth in crushing and grinding food. Bilateral extraction of all maxillary and mandibular molars removed most posterior occlusal support and would be expected to reduce the effective occlusal contact area, alter mandibular movement, impair bolus formation, and decrease the production of fine food particles.^2^^,^^5^, ^6^, ^7^ The exceptionally large standardized effect should nevertheless be interpreted cautiously. It reflects near-complete separation between two small groups with low within-group variability and may have been amplified by the severity of the intervention, attrition during the experiment, and the limited precision of standardized effect estimates derived from small samples. The effect-size estimate should therefore be interpreted together with the absolute mean difference and its confidence interval rather than as a precise estimate of the population effect.

The reductions in BDNF and TrkB immunoreactivity are consistent with attenuation of an activity-dependent neurotrophic environment. Oral afferent input from the periodontal ligament, oral mucosa, and masticatory muscles is transmitted through trigeminal and related neural networks that may indirectly influence hippocampal activity.^8^^,^^15^ BDNF contributes to neuronal survival, dendritic growth, synaptogenesis, and long-term potentiation, whereas TrkB mediates downstream signaling involved in activity-dependent neural plasticity.^14^, ^15^, ^16^, ^17^, ^18^ However, the present immunohistochemical findings do not establish changes in BDNF protein concentration, BDNF or NTRK2 transcription, TrkB phosphorylation, or activation of downstream ERK, Akt, and CREB signaling. The results should therefore be interpreted as differences in immunoreactivity rather than direct evidence of reduced activation of the complete BDNF–TrkB signaling pathway.

The findings are broadly concordant with previous reports indicating that juvenile tooth loss or reduced mastication may alter hippocampal neurogenesis, BDNF/TrkB-related signaling, synaptic structure, and memory-related behavior.^8^, ^9^, ^10^, ^11^^,^^24^, ^25^, ^26^ The pooled correlations in the present study further showed that animals with higher MPI values tended to have higher hippocampal neurotrophic scores. These correlations should be regarded as exploratory because the control and extraction groups occupied largely separate ranges of both variables. Consequently, the coefficients may have been driven primarily by between-group separation rather than a continuous within-group relationship. The findings therefore do not establish that reduced masticatory performance mediates the association between posterior tooth extraction and hippocampal BDNF or TrkB immunoreactivity. Accordingly, the pooled coefficients should be interpreted primarily as reflecting separation between the experimental groups rather than as evidence that individual animals with better masticatory performance necessarily had higher BDNF or TrkB immunoreactivity within each group.

Several alternative or complementary mechanisms remain plausible. Posterior tooth extraction may reduce trigeminal afferent stimulation, but the intervention may also produce anesthesia-related effects, surgical stress, postoperative pain, local inflammation, reduced food intake, weight loss, dehydration, and systemic metabolic changes.^15^^,^^24^^,^^27^ The extraction group received a soft diet during the first three postoperative days, whereas the control animals continued to receive standard pellets. Because the study did not include sham-extraction or anesthesia-only control groups and did not longitudinally quantify food intake, body weight, corticosterone, inflammatory biomarkers, pain-related behavior, hydration, or metabolic status, the observed hippocampal differences cannot be attributed exclusively to reduced mastication. The temporary difference in postoperative diet should therefore also be regarded as a potential confounding factor.

Attrition represents an important interpretive concern. Seven of the 24 enrolled animals did not reach the study endpoint, corresponding to an overall attrition rate of 29.2%. This reduction in sample size decreased statistical precision and may have affected the comparability of the final groups. If attrition was related to the extraction procedure or to postoperative physiological changes, the animals remaining at the endpoint may have represented a selected subset of the initially enrolled sample. Such selection could influence group means, within-group variability, and standardized effect estimates. Because the available study documents did not provide a fully consistent account of the timing, group distribution, clinical circumstances, and causes of all deaths, cause-specific interpretations of attrition remain limited. The findings should consequently be interpreted as complete-case results from animals that reached the experimental endpoint.

The available study documentation described allocation in a 1:1 ratio but did not provide sufficient detail regarding the method used to generate the allocation sequence, the individual responsible for sequence generation, or whether formal allocation concealment was implemented. This limits assessment of the risk of selection bias. Chewing-residue samples were assigned coded identifiers before MPI processing, and immunohistochemical scoring was independently performed by two observers who were blinded to group allocation. However, complete blinding of the MPI assessor and statistical analyst could not be independently verified from the available records. The possibility of detection or analysis bias therefore cannot be excluded.

The bilateral extraction of all maxillary and mandibular molars represents a severe experimental model of posterior occlusal deprivation rather than a direct reproduction of the usual clinical pattern of premature posterior tooth loss in children. This standardized intervention produced a substantial reduction in posterior occlusal support and measurable masticatory impairment across animals. In pediatric populations, premature tooth loss is generally less extensive and more heterogeneous with respect to the number of teeth involved, timing, underlying disease, dietary adaptation, and occlusal compensation. The magnitude of the functional and hippocampal differences observed in this model should therefore not be directly extrapolated to children with premature loss of individual primary molars.

Translation of these findings to pediatric dentistry should remain conservative. The present experiment did not evaluate hippocampus-dependent behavior and does not demonstrate cognitive impairment in children. Moreover, associations between tooth loss and cognitive outcomes in human populations are vulnerable to confounding by socioeconomic conditions, diet, nutrition, systemic health, education, and access to dental care.^28^ The findings support established clinical priorities, including prevention of caries-related posterior tooth loss, preservation of restorable primary molars, maintenance of functional posterior occlusal support, and appropriate space management when extraction is unavoidable.^2^^,^^5^^,^^29^, ^30^, ^31^ They do not justify using dental status, masticatory performance, or assumptions regarding BDNF signaling to predict an individual child's cognitive development.

The study has several additional limitations. Only juvenile male rats were included, limiting generalizability across sex. The post-test-only design precluded assessment of baseline values and longitudinal changes. BDNF and TrkB were assessed using different semi-quantitative scoring systems, preventing direct numerical comparison between the two markers. Interobserver reliability was not reported in the available study records. Hippocampus-dependent learning and memory were not assessed using behavioral tests. Molecular validation using qPCR, Western blotting, ELISA, phosphorylated TrkB measurements, or downstream signaling assays was not performed. In addition, the resource-equation approach used to determine enrollment did not constitute a formal a priori power calculation and did not provide a prespecified estimate of statistical precision. Finally, analysis was restricted to animals that completed the experiment, and the effect of attrition on the observed estimates could not be formally evaluated. An important additional limitation concerns the reproducibility of the BDNF scoring procedure. Although field-level staining observations were recorded using a binary negative/positive classification, the original algorithm used to transform these observations into the reported 0-10 subregional scores could not be recovered from the retained study records. Consequently, the exact BDNF scoring procedure cannot be independently reproduced. The BDNF findings should therefore be interpreted as exploratory semi-quantitative observations and require confirmation using a fully predefined scoring system and quantitative molecular methods.

Future experiments should use preregistered and adequately powered designs with clearly documented sequence generation, allocation concealment, and outcome-assessor blinding. Sham-extraction and anesthesia-only control groups should be included to distinguish the consequences of loss of posterior occlusal function from those of anesthesia, tissue injury, pain, and postoperative stress. Both male and female animals should be studied, with longitudinal monitoring of body weight, food intake, pain-related behavior, corticosterone, inflammatory biomarkers, and metabolic status. Hippocampus-dependent behavioral testing should be combined with region-specific molecular validation of BDNF–TrkB and ERK/Akt/CREB signaling.^32^^,^^33^ Presentation of individual-animal data, examination of within-group associations, and independent replication will be required before proposing mediation or a clinically relevant oral–brain mechanism. Translational studies in children should initially focus on objectively measured masticatory performance, dietary adaptation, nutrition, oral motor function, and developmental dental outcomes rather than inferring cognitive effects directly from molecular findings in animals.

## 5 Conclusion

Bilateral molar extraction during growth in juvenile male Wistar rats was associated with lower masticatory performance and reduced hippocampal BDNF and TrkB immunoreactivity. The pooled correlations were exploratory and may largely reflect between-group separation; they do not establish within-group biological relationships, mediation, or causality. Substantial attrition, the absence of sham controls, limited reproducibility of the transformation used to derive the BDNF 0–10 subregional scale, and unmeasured postoperative nutritional, inflammatory, and stress-related factors require cautious interpretation. Larger, independently replicated behavioral and molecular studies are needed before mechanistic or clinical cognitive implications can be inferred.

## Patient's/guardian's consent

Not applicable. This was an animal experimental study and did not involve human participants.

## Ethical approval

The animal protocol was approved by the Ethics Committee of the Faculty of Dentistry, Hasanuddin University Dental Hospital, Hasanuddin University (No. 298/KEPK-FKG-RSGMP-UH/EA/X/2025).

## Related manuscript disclosure

JOBCR-D-26-00607R1 is a companion report from the same parent animal experiment and evaluates GFAP and synaptophysin outcomes. The shared cohort and animal-level MPI dataset are explicitly disclosed in Methods Section 2.1; no GFAP or synaptophysin outcome data are reported in the present manuscript.

## Ethical clearance

This study was approved by the Ethics Committee of the Faculty of Dentistry, Hasanuddin University Dental Hospital, Hasanuddin University (No. 232/KEPK-FKG-RSGMP-UH/EA/X/2025).

## Data availability

The datasets generated and/or analyzed during the current study are available from the corresponding author on reasonable request.

## Author contributions

Conceptualization and methodology: Harun Achmad, Dharli Syafriza.

Data collection and experimental procedures: Sri Ratna Rahayu.

Statistical analysis and data interpretation: Harlina, Andi Annisa Eka Aprilda, Andi Aliyah Nurul Syaikah, Nurul Mutamainnah, Fitri Apriliani, and Yunita Dyah Puspita Santik.

Writing - original draft: Harun Achmad, Dharli Syafriza.

All authors reviewed, approved, and agreed to the final version of the manuscript.

## Declaration of generative AI and AI-assisted technologies in the manuscript preparation process

During the preparation of this work, the authors used AI for language refinement and manuscript formatting assistance. After using this tool, the authors reviewed and edited the content as needed and take full responsibility for the content of the published article. No AI tools were used for data generation, data analysis, or scientific interpretation.

**Human and animal rights statement**

**Full statement for title page/Editorial Manager**

Human and Animal Rights Statement: This study did not involve human participants, human tissue, identifiable human data, patient descriptions, clinical photographs, radiographic images, or histopathologic images from patients; therefore, human informed consent, consent to participate, and consent for publication were not applicable. The animal experimental protocol and all procedures involving juvenile male Wistar rats (*Rattus norvegicus*) were reviewed and approved by the Ethics Committee of the Faculty of Dentistry, Hasanuddin University Dental Hospital, Hasanuddin University (No. 232/KEPK-FKG-RSGMP-UH/EA/X/2025). All animal procedures were performed in accordance with the approved institutional animal ethics protocol and relevant ethical principles for the care and use of laboratory animals. Animals were housed under controlled environmental conditions, monitored for signs of illness or distress, anesthetized for posterior tooth extraction, and euthanized at the study endpoint according to the approved protocol. The authors made efforts to minimize animal suffering and to use the minimum number of animals necessary to achieve the scientific objectives.

**Blinded statement for anonymized manuscript, if requested**

Human and Animal Rights Statement: This study did not involve human participants, human tissue, identifiable human data, patient descriptions, clinical photographs, radiographic images, or histopathologic images from patients; therefore, human informed consent, consent to participate, and consent for publication were not applicable. The animal experimental protocol and all procedures involving juvenile male Wistar rats (*Rattus norvegicus*) were reviewed and approved by an institutional ethics committee (approval details blinded for review). All animal procedures were performed in accordance with the approved institutional animal ethics protocol and relevant ethical principles for the care and use of laboratory animals. Animals were housed under controlled environmental conditions, monitored for signs of illness or distress, anesthetized for posterior tooth extraction, and euthanized at the study endpoint according to the approved protocol. Efforts were made to minimize animal suffering and to use the minimum number of animals necessary to achieve the scientific objectives.

## Sources of funding

This research is funded by the Indonesian Endowment Fund for Education (LPDP) on behalf of the Indonesian Ministry of Higher Education, Science and Technology and managed under the EQUITY Program (Contract No. 4307/B3/DT.03.08/2025 and 47103/UN4.1/PM.01.01/2025).

## Declaration of competing interest

The authors declare that they have no known competing financial interests or personal relationships that could have appeared to influence the work reported in this paper.
